# Iteration Bayesian Reweighed Algorithm for Optical Carrier-Based Microwave Interferometry Sensing

**DOI:** 10.3390/s20113079

**Published:** 2020-05-29

**Authors:** Yuxiao Li, Ciming Zhou, Dian Fan, Sijing Liang, Li Qian

**Affiliations:** 1 National Engineering Laboratory for Fiber Optic Sensing Technology, Wuhan University of Technology, Wuhan 430070, China; lyx0313@whut.edu.cn (Y.L.); zcm@whut.edu.cn (C.Z.); lsj123@whut.edu.cn (S.L.); 2 School of Information Engineering, Wuhan University of Technology, Wuhan 430070, China; 3 Department of Electrical and Computer Engineering, University of Toronto, Toronto, ON M5S 3G4, Canada; l.qian@utoronto.ca

**Keywords:** optical carrier-based microwave interferometer, fiber optics sensors, microwave photonics, bayesian estimation, frequency fluctuation

## Abstract

This paper proposes a novel iteration Bayesian reweighed (IBR) algorithm to obtain accurate estimates of a measurement parameter that uses only a few noisy measurement data. The method is applied to optimize the frequency fluctuation in an optical carrier-based microwave interferometry (OCMI) system. The algorithm iteratively estimates the frequency of the S-parameter valley point by collecting training samples to rebalance the weights between prior samples, which reduces the impact of noise in the system. Simulation shows that the estimated result of this algorithm is closer to the true value than that of the maximum likelihood estimation (MLE) using the same amount of measured data. Under the influence of system noise, this algorithm optimizes the frequency fluctuation of the S-parameter and reduces the impact of individual measured data. In this study, we applied the algorithm in the strain sensing experiment and compared it with the MLE. When axial strain changes 240 με, the IBR algorithm yields a deviation of 36 με, which is a significant reduction from 138 με (using the MLE method). Moreover, the average error rate decreases from 25% to 3% (with the MLE method), suggesting that the linear fitting degree of the estimated results and accuracy of the system are improved. Moreover, the algorithm has a wide range of applicability, for it can handle different application models in the OCMI system and the systems with frequency fluctuation problems.

## 1. Introduction

Optical carrier-based microwave interferometry (OCMI) has better stability than the traditional optical interferometry because the microwave wavelength is much longer than the optical wavelength [1]. OCMI uses a microwave-modulated optical carrier with an interferometer-based sensor that converts an optical path length change (due to strain or temperature) into a relative frequency shift in the microwave interference spectrum, which is subsequently measured using a commercial vector network analyzer (VNA) [2]. Interference fringe reconstruction in microwave domain has the advantages of high visibility [3,4] convenient demodulation, and long coherence length [5].

**Figure 1:**
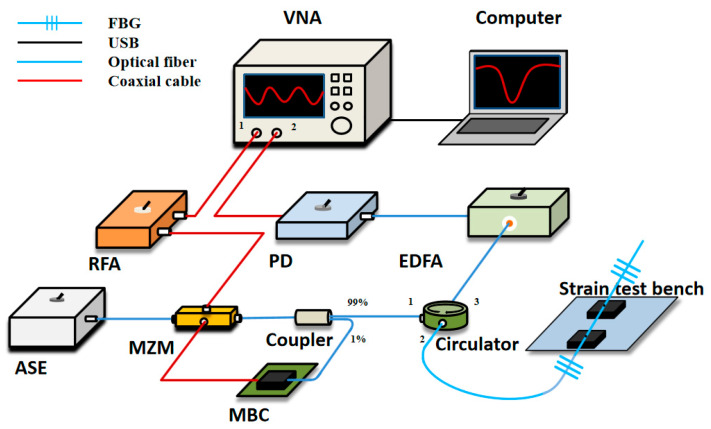
Schematic of the optical carrier-based microwave interferometry (OCMI) system configuration for concept demonstration. VNA: vector network analyzer. ASE: amplified spontaneous emission light source. MZM: Mach-Zehnder modulator. RFA: radio frequency amplifier. MBC: MZM’s bias controller. EDFA: erbium-doped fiber amplifier. PD: high-speed photodetector.

A typical OCMI setup is shown in Figure 1. The optical carrier from an amplified spontaneous emission (ASE) is modulated using a frequency-swept microwave signal via the Mach-Zehnder modulator (MZM) before being sent to the interferometer-based sensor. The signal reflected by the interferometer is amplified using an erbium-doped fiber amplifier (EDFA) and converted into an electrical signal using a high-speed photodetector (PD).

Huang et al. presented a microwave interrogated sapphire fiber Michelson interferometer for high temperature sensing and verified the stability of the system at room temperature at 4300 MHz, with a frequency deviation of ±30 kHz, corresponding to a temperature variation of ±0.5 °C [6]. However, to improve the measurement accuracy, it is necessary to increase the frequency range of the VNA, which determines the measuring resolution of the system. The larger it is, the higher the resolution [5]. However, because the interference fringe spectrum takes the VNA frequency as the abscissa, the VNAs higher measuring frequency causes the interference fringe frequency fluctuation to increase due to system noise. There are many sources of system noise such as ASE, PD, EDFA, and VNA. ASE causes relative intensity noise and phase noise [7,8], while PD causes dark current noise, thermal noise, and the shot noise [9]. EDFA causes excess noise [10]. In addition, even if the VNA has been calibrated first, there will still be residual error [11]. The presence of these noises results in frequency errors in the S-parameters obtained using the VNA, which leads to uncertainties in the measured optical path length change. Researchers are required to find a method to solve the frequency fluctuation problem in OCMI.

Similar to the frequency fluctuation in OCMI, the frequency offset caused by the Doppler effect in orthogonal frequency division multiplexing (OFDM) is shown as the frequency randomly fluctuates with changes in the external environment [12]. OFDM is an efficient signaling scheme for digital communications. A variety of algorithms have been used to reduce the frequency offset and obtain the real frequency, such as matching between the transmitter and the receiver. Moose, P.H. [12] discusses the uncertainties of frequency offset on OFDM performance, using an MLE algorithm to estimate the actual frequency offset. This algorithm generates extremely accurate estimates even when the offset is far too great to demodulate the signal. Lin, D.D. et al. [13] derive the maximum a posteriori (MAP) channel estimator for phase noise and frequency offset. Zou, Q. et al. [14] propose an iterative algorithm to jointly estimate the channel response with phase noise and frequency offset. Balogun, M.B. et al. [15] propose an effective estimation scheme based on the ML approach. The scheme mitigates and compensates the undesirable effect of carrier frequency offset on the coherent optical OFDM system. Further, the ML approach is simplified [16]. In general, these approaches in OFDM system show that an effective estimation method can improve measurement accuracy in the presence of noise.

As for estimation methods, the Bayesian theory has relevant applications in many fields. Halimi A et al. [17] propose a Bayesian iteration algorithm to estimate the smooth Gaussian noise signal by conducting experiments on satellite altimetry data. This leads to a good de-noising effect on both the synthetic signal and the actual signal. Sanger, T.D. [18] proposes a novel recursive algorithm for the on-line Bayesian iteration of the EMG signal surface. The primary advantage of this algorithm is the possibility of smooth output signals without eliminating the possibility of sudden and large value changes. Xiang, Y. et al. [19] propose a general framework via the recursive Bayesian state estimation for single target tracking in cognitive networks of radars. However, to the best of our knowledge, these methods have not been applied to the OCMI system.

Based on the above methods and in accordance to OCMI system characteristics, this paper develops a novel iteration Bayesian reweighed (IBR) algorithm, which is an algorithm for rebalancing the weights used for samples and priors through iterations, using Bayesian theory. We applied the algorithm to an OCMI system to reduce the impact of system noise on the frequency fluctuation, obtaining stable and accurate measurement results with fewer measurements.

## 2. Principle

The schematic of the OCMI system is shown in Figure 1, which includes a strain test bench for experimental validation. The continuous light-wave from the ASE is modulated using a microwave signal, through the MZM. The microwave signal is generated from VNA port 1 and sent to the MZM after being amplified using a radio frequency amplifier (RFA). The MZM is modulated at a quadrature point using the MZM’s bias controller (MBC). This is achieved through sending 1% of the MZM output to MBC’s controller. Moreover, 99% of the MZM output is sent to port 1 of the circulator and the output (port 2), which is connected to a pair of fiber Bragg gratings (FBGs). The fiber between the two gratings can be used as a strain sensor. The signals reflected by the gratings go to port 3 of the circulator and pass through the EDFA. Then the PD converts the optical signal into an electric signal, which finally goes to VNA port 2. The signal obtained from VNA are demodulated and optimized using a computer to obtain the final strain sensing results.

The signal presented on the VNA is the ratio of the electric signal from port 2 to the signal from port 1, which is called the S-parameter. The ideal S-parameter (S) can be expressed as [2]:

(1) $$S=10\mathrm{lg}\left(A^{2}J_{1}(m)\sqrt{2+2\cos2\pi f_{D}n\frac{\left(\varepsilon +1\right)L_{s}}{c}}\right)$$

where A is the optical signal amplitude, n is the refractive index of the optical fiber, and c is the propagation speed of light. $J_{1}(m)$ is the Bessel function of the first order and the first class. $f_{D}$ is the frequency of microwave signal. ε is the applied strain and $L_{s}$ is the length of the optical fiber sensor.

The free spectral range (FSR) is defined as the frequency difference between two adjacent extrema of the S-parameter and is inversely proportional to the optical path difference (OPD) of the fiber optic interferometer sensor [2]:

(2) $$FSR=\frac{1}{OPD}=\frac{c}{nL_{s}}$$

Similar to optical interference, the S-parameter spectrum of optical microwave interference can demodulate and be used to extract the sensor’s OPD. The VNA takes the sweeping frequency of the microwave signal as the abscissa, when the OPD changes due to strain, the S-parameter spectrum will shift. By measuring the frequency shift of an extremum (a valley point) of the S-parameter, the OPD change can be measured and the strain change can be deduced. According to Equations (1) and (2), the applied strain ε can be expressed as:

(3) $$\varepsilon =\frac{\Delta OPD}{OPD}=\frac{\Delta f}{f}$$

where f is the frequency of the valley point of S-parameter and Δf is the frequency shift of the valley point. It can be found from Equation (3) that the applied strain ε is directly related to the valley point frequency f and the frequency shift Δf. The accuracy of f and Δf demodulation exactly reflects the accuracy of the system.

However, the S-parameter in the presence of noise (*S_WN_*) can be expressed as:

(4) $$S_{WN}=10\mathrm{lg}\left(\left(A^{2}+\frac{A_{N}}{A_{m}}\right)J_{1}(m)\sqrt{2+2\cos2\pi f_{D}n\frac{\left(\varepsilon +1\right)L_{s}+W}{c}+\varphi }\right)$$

where $A_{m}$ is the microwave signal amplitude and $A_{N}$ is the amplitude variation caused by noise. W is electric delay term and φ is the phase variation caused by noise. $A_{N}$ and φ are a mixture of noise produced by ASE, EDFA, and PD in the system. W is caused by the coaxial cable used to connect optical fiber sensing part and the circuit demodulation part of the system, as well as VNA factors. The uncertainty and instability of W and φ in Equation (4) lead to the instability of Δf in Equation (3), influencing f to cause the instability of the valley point of the S-parameter, which is presented as the phenomenon of frequency fluctuation. These factors lead to the inaccuracy of the final measured strain and the results of different measured data are inconsistent because the FSR is different. In order to get an accurate and stable measurement result, we need hundreds of measurements to filter out results, which is inconvenient when the experimental data is incomplete.

Inspired by similar methods [12,13,14,15,16,17,18,19], we refer to the theory of Bayesian estimation and use an effective estimation method to improve accuracy in the presence of noise. Since only the frequency of the valley point of the S-parameter is required for demodulation, all of the parameters in the algorithm are related to the frequency of the valley point of S-parameter. All collected samples in the S-parameter are independent of each other.

According to the Bayesian estimation theory, under the condition of existing, the measured data of the S-parameter with noisy version is ($S_{WN}\left(x\right),x=1,\;2,\ldots \;,\;n$), the probability of training sample of S-parameter is ($S_{TS}\left(x\right),x=1,\;2,\ldots ,\;m$), and occurrence can be expressed as [20]:

(5) $$\begin{array}{cc} p\left(S_{\mathrm{TS}}(x)|S_{WN}(x)\right) & =\int p\left(S_{\mathrm{TS}}(x)|\theta ,S_{WN}(x)\right)p\left(\theta |S_{WN}(x)\right)d\theta \\ & =\int p\left(S_{\mathrm{TS}}(x)|\theta \right)p\left(\theta |S_{WN}(x)\right)d\theta \end{array}$$

where θ is a prior parameter of f and $p\left(S_{TS}(x)|\theta \right)$ is a likelihood function. $p\left(\theta |S_{WN}(x)\right)$ is the posteriori probability of θ under the condition of existing $S_{TS}\left(x\right)$.

Since θ and $p\left(S_{TS}(x)|\theta \right)$ are unknown, in order to get the estimated result of f, we need to estimate the prior information; θ and $p\left(S_{TS}(x)|\theta \right)$ would be estimated first.

The training samples $S_{TS}(x)$ are collected before the strain was applied to the sensor for estimating the likelihood function $p\left(S_{TS}(x)|\theta \right)$. Assuming $S_{TS}(x)$ satisfies the Gaussian distribution with mean value $\mu_{r}$ and variance $\sigma_{r}{}^{2}$, its likelihood function can be expressed as:

(6) $$p(S_{TS}(x)|\theta )=\prod_{x=1}^{n}\frac{1}{\sqrt{2\pi }\sigma_{r}}\exp[-\frac{{\left(S_{TS}(x)-\mu_{r}\right)}^{2}}{2\sigma_{r}{}^{2}}]$$

When $S_{TS}\left(x\right)$ is large enough, the MLE results of $\mu_{r}$ and $\sigma_{r}{}^{2}$ can be regarded as the true probability density function of ideal f, because it is asymptotically unbiased and asymptotically consistent [21]. In the strain sensing experiment of OCMI, the only thing changed by the different strain is the sensor length, and it will not affect the noise in Equation (4). Therefore, we can reasonably suppose that there is a common variance $\sigma_{r}{}^{2}$ of measured data $S_{WN}(x)$ after each strain.

To estimate the prior parameter θ, we collected groups of the measured training sample for the S-parameter after various amounts of strain were applied ($S_{TA}\left(x\right),x=1,\;2,\;\ldots ,\;k$). Assuming the prior parameter θ satisfies the Gaussian distribution with mean value $\mu_{n}$ and variance $\sigma_{n}{}^{2}$, its likelihood function can be expressed as:

(7) $$p(\theta )=\prod_{x=1}^{n}\frac{1}{\sqrt{2\pi }\sigma_{n}}\exp[-\frac{{\left(S_{TA}(x)-\mu_{n}\right)}^{2}}{2\sigma_{n}{}^{2}}]$$

The MLE results of θ can be obtained by taking logarithm of likelihood function and making partial derivative equal to zero.

According to the Bayesian theory, the posteriori probability of θ under the condition of existing $S_{WN}\left(x\right)$ can be expressed as:

(8) $$p\left(\theta |S_{WN}(x)\right)=\frac{p\left(S_{WN}(x)|\theta \right)p\left(\theta \right)}{\int p\left(S_{WN}(x)|\theta \right)p\left(\theta \right)d\theta }$$

where $p\left(S_{WN}(x)|\theta \right)$ is a likelihood function similar to $p\left(S_{TS}(x)|\theta \right)$.

Next, once we get all prior information using Equations (6)–(8), the estimated results of f can be expressed as:

(9) $$\hat{f}=\arg\underset{\theta }{\max}p\left(\theta |S_{WN}(x)\right)$$

However, the estimated results depend largely on the prior information because it determines the weights of the measured data. Moreover, the prior information is random when the number of the training sample $S_{TA}(x)$ is small. Even with a lot of optimization, the preciseness of the estimated results cannot be ensured in the absence of the reliable prior parameter. The recursive Bayesian estimation [22] can be used to obtain the estimated value without accurate prior information, but in order to get stable estimated results, it needs a lot of sample training, which does not meet the requirement of our OCMI strain experiment, which is to get the accurate estimated value with only a few measurements. In our system, what we need is the true value of f after each measurement. An accurate and stable prior parameter can make the estimated result closer to the real value and can get a relatively accurate and stable estimated value in a small number of measured data. In order to obtain an accurate prior parameter for OCMI system’s greater stability, we designed an iterative algorithm to compensate for the lack of prior information accuracy.

The main idea of our algorithm is to reduce the influence of inaccurate prior parameters on the estimation results by reweighting prior parameters and measured data in an iterative way based on Bayesian theory. We follow a two-step approach to estimate f.

First, we estimate the prior information, which we refer to as the estimation step (E-step). We assume that the estimated results of f satisfies the Gaussian distribution with a mean value μ and variance $\sigma^{2}$. Using one group of measured data ($S_{WN}\left(x\right),x=1,\;2,\;\ldots ,\;n$), the estimated results, according to Equation (9), can be expressed as,

(10) $$\hat{\mu }=\frac{\sigma_{n}^{2}S_{WN}(1)+\sigma_{r}^{2}\mu_{{}_{n}}}{\sigma_{r}^{2}+\sigma_{n}^{2}}$$

(11) $$\hat{\sigma^{2}}=\frac{\sigma_{r}^{2}\sigma_{n}^{2}}{\sigma_{r}^{2}+\sigma_{n}^{2}}$$

The second step features the iterative algorithm, which is used to rebalance the weight between the prior and measured data, which can be called the reweighing step (R-step). The algorithm first uses the estimated results in Equations (10) and (11) to substitute the prior information in Equation (7) before participating in a new round of estimation calculations.

The core equation of the iterative algorithm can be expressed as:

(12) $$\hat{\sigma^{2}}(x)=\frac{\sigma_{r}^{2}\sigma^{2}(x-1)}{\sigma_{r}^{2}+(x-1)\sigma^{2}(x-1)}$$

(13) $$\hat{\mu }(x)=\left(\frac{\sum_{i=1}^{x}S_{WN}(i)}{\sigma_{r}^{2}}+\frac{\overset{\wedge }{\mu }(x-1)}{\sigma^{2}(x)}\right)\frac{\sigma_{r}^{2}\sigma^{2}(x)}{\sigma_{r}^{2}+x\sigma^{2}(x)}$$

By substituting the measured data into the equation, the variance in Equation (12) becomes smaller and the estimated value in Equation (13) fluctuates less. From Equations (12) and (13) we can deduce:

(14) $$\underset{x\to \infty }{\lim}\hat{\sigma^{2}}(x)=\underset{x\to \infty }{\lim}\frac{\sigma_{r}^{2}\sigma^{2}(x-1)}{\sigma_{r}^{2}+x\sigma^{2}(x-1)}=0$$

(15) $$\underset{x\to \infty }{\lim}\hat{\mu }(x)=\underset{n\to \infty }{\lim}\frac{1}{n}\sum_{x=1}^{n}S_{WN}(x)$$

It can be concluded that, according to Equations (14) and (15), the MLE and Bayesian estimation are equal when the number of measured data approaches infinity. Finally, an estimated value with high reliability is obtained after all measured data are traversed.

(16) $$\hat{f}=\hat{\mu }(n)$$

## 3. Algorithm and Simulation

In this section, we propose a novel IBR algorithm according to the above theory.

**Figure 2:**
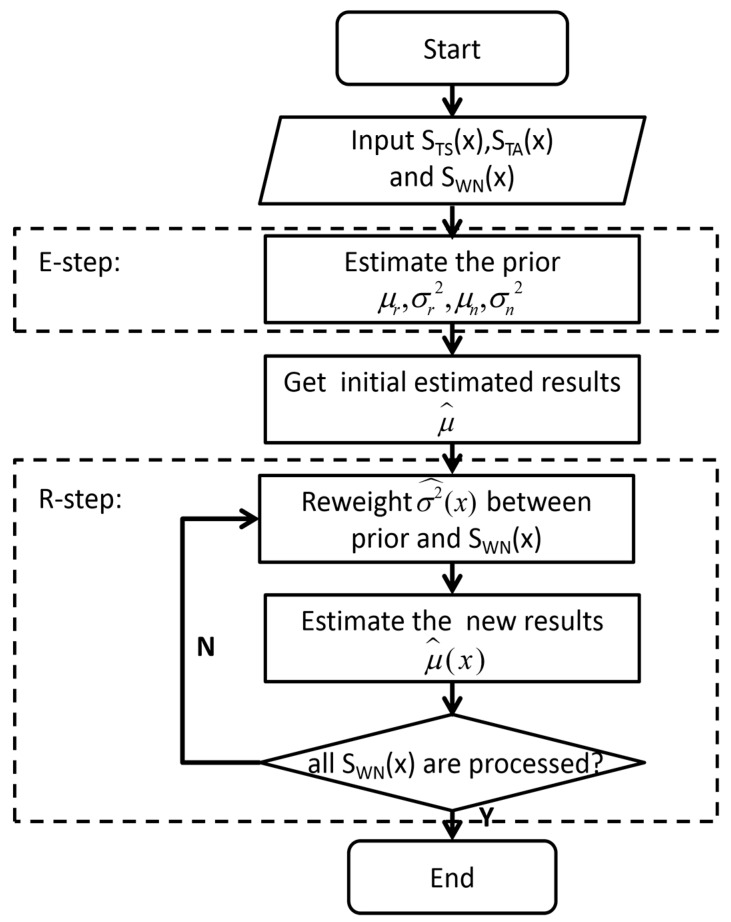
Iteration Bayesian reweighed (IBR) algorithm process.

The flow chart of the algorithm is as follows:

In Figure 2, the IBR algorithm obtains the estimated prior $\mu_{r},\sigma_{r}^{2},\mu_{{}_{n}},\sigma_{n}^{2}$ by using the training samples $S_{TS}\left(x\right)$ and $S_{TA}\left(x\right)$ in E-step. According to Equation (10), the initial estimated value is gained, which is $\hat{\mu }$. In order to make the estimated result closer to the true value and more reliable, the algorithm was implemented by iterative reweighting in the R-step. After getting the initial estimated value, the measured data $S_{WN}\left(x\right),x=1,\;2,\;\ldots ,\;n$ were updated continuously by reweighing the prior and the data according to Equation (12). The algorithm made the accurate prior information have more weight and the fluctuation range of the result smaller. With more accurate prior information, the new estimated result $\hat{\mu }(x)$, according to Equation (13), tends to be stable and closer to the truth value. In this way, the algorithm can optimize the estimated result while ensuring its rigor and reliability, reducing the possibility of an inaccurate estimated result caused by a prior parameter inaccuracy. Finally, an estimated value $\hat{f}=\hat{\mu }(n)$ with high reliability in Equation (16) was obtained after all measured data are traversed.

**Figure 3:**
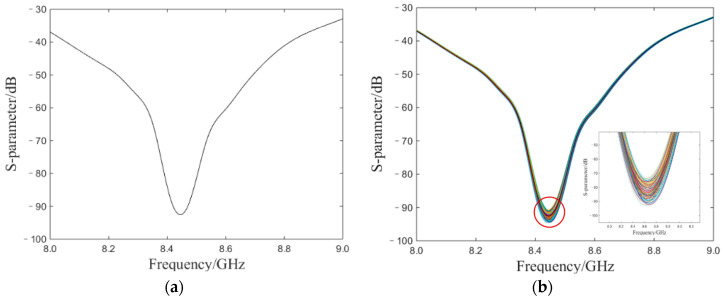
(**a**) The simulated S-parameters spectrum of signal frequency domain. (**b**) The simulated S-parameters of simulations with noise.

To validate the IBR algorithm given above, we explored the performance of MLE and IBR by applying them to simulated S-parameters in an OCMI system. In the simulated system, the simulated sensor length was 0.15 m, the microwave sweep range was from 8 GHz to 9 GHz, and the number of sampling points was 100,000. We designed three simple Gaussian white noise $A_{N}$, W, and φ in Equation (1) with a variance of 0.001. Although we tried to restore the real system values as much as possible, the simulated Gaussian white noise could not completely reproduce the actual noise. Though the white noise was different from the noise in the actual S-parameter, the simulated S-parameter also exhibited valley point frequency fluctuation, which was used for the verification of the novel algorithm. Figure 3a shows the simulation of an ideal S-parameter without noise and the frequency of the original S-parameter peak point is 8444.78 MHz. Figure 3b contains 500 interference patterns of S-parameters under the influence of noise. According to Equation (3), the amplitude of S-parameter did not participate in the calculation of demodulation. The frequency value of the valley point is the data measured. MLE and IBR were used to deal with the simulated S-parameter, respectively.

**Figure 4:**
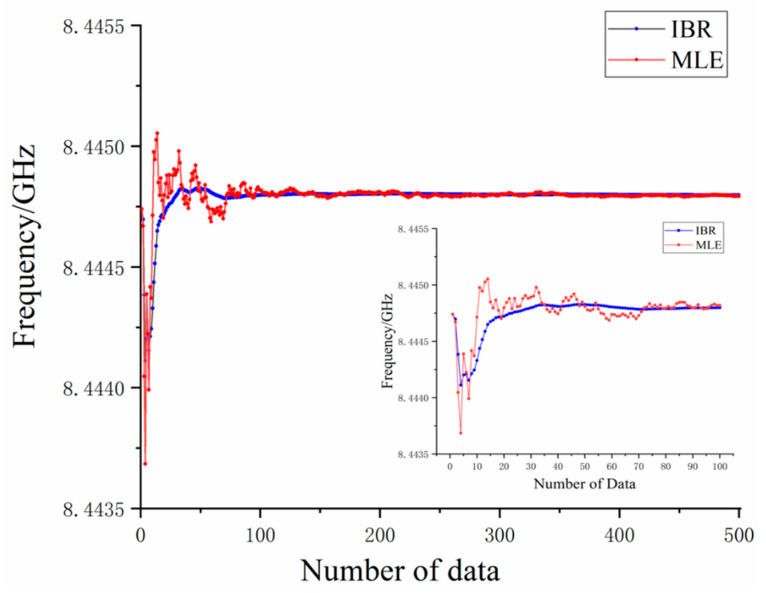
Estimated frequency at the valley of simulation signals.

In order to show the effect of IBR and MLE, the estimated results are shown according to the increase of simulated data volume. Figure 4 shows how both algorithms have a similar trend for the S-parameter estimation results, which start around 8444.75 MHz, decreases at the third set of simulated data, fluctuates up and down as the simulated data increases, and finally stays around 8444.79 MHz. This is consistent with the ideal value. Comparing to MLE, the curve fluctuation range of simulated S-parameter frequency estimated by the IBR algorithm is much smaller and the curve of IBR becomes smooth when the number of simulated data increases. In the initial stage, which is around 20 sets of simulated data, it reached a relatively stable value. It clearly shows that when the data volume reaches 300, the curves of the estimated results by the two algorithms are basically the same, which proves Equation (15).

From the above simulation’s results, it is obvious that, compared to the MLE, the estimated result of IBR can reach a stable value in a short amount time. There are large frequency fluctuations in the MLE results when the amount of simulated data is insufficient; there are not enough data to support a baseline and there are simulated data with a huge frequency deviation, which affects the estimated value. However, for IBR, the problem is well mitigated. The random data did not affect the estimated value because we reweighed the prior samples in an iterative way. As the iteration goes on, the weight of the simulated data became smaller and the estimated value became stable without much disturbance from large frequency deviation data. IBR quickly achieved an accurate and stable estimated value. In the case of 20–30 simulated data, it reached the effect of MLE using 300 simulated data. The simulation result verified the core idea of the algorithm proposed and we unearthed the benefits of the IBR’s stability, which is exactly what we needed from our OCMI system.

## 4. Experiment and Discussion

According to the simulation results, we carried out strain sensing experiments in OCMI to verify the algorithm further. The experimental diagram is shown in Figure 1, where the VNA model was ZVA67 (Rohde and Schwarz, Munich, Germany). The number of sampling points of the receiver was set to 60,000. The microwave bandwidth was set to be 1 GHz (from 8 GHz to 9 GHz). The minimum resolvable frequency shift of VNA was equal to the sweep frequency range divided by the number of sampling points, which was about 16,666 Hz. The speed of one sweep was about 2 s. The ASE light source had an output wavelength range of 1527–1604 nm, a 3 dB bandwidth of 77 nm, and a power of 10 dBm of continuous light. RFA and EDFA both operated at a gain of 20 dB. The bandwidth of the PD was 10 GHz. The sensor in the OCMI system was the fiber between the two ultra-weak fiber Bragg gratings whose reflectivity was around –30 dB and the sensor length was 15 cm. We intended to use the OCMI system to measure the fiber strain changes between the two ultra-weak gratings. To prevent the gratings themselves from being affected by strain during the experiment, the optical fiber was bonded at both ends of the grating to the strain test bench.

**Figure 5:**
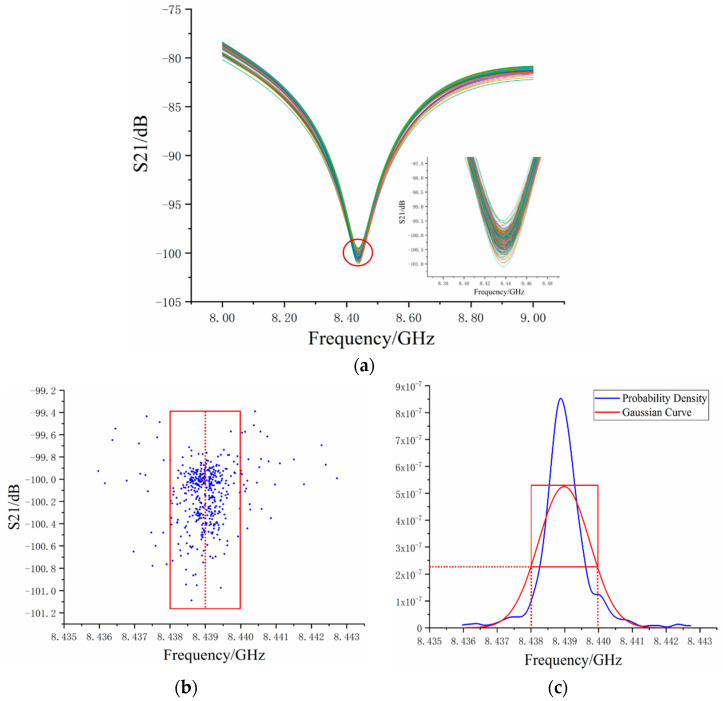
(**a**) Spectrum interferogram of S-parameters of 437 sets of measured data. (**b**) Coordinates of S-parameter valley of 437 sets of measured data. (**c**) Probability density and Gaussian fitting of valley coordinate frequency.

In order to apply the algorithm in the actual sensing experiment, after the system had been setup we collected the training sample $S_{TS}\left(x\right)$ for the $\mu_{r}$ and $\sigma_{r}{}^{2}$. Through the correlation calculation of Equation (5), we obtained the estimated prior parameter, which was reasonably assumed to be the common variance of the ideal S-parameter. Figure 5a contains 470 sets of measured S-parameters. There was too much data and the variations were not obvious enough in Figure 5a. In order to observe the frequency fluctuation more clearly, the valley point coordinates of each measured data group are displayed in Figure 5b. Figure 5b shows that the frequency of the valley point ranges from 8435 MHz to 8443 MHz, most of which were between 8438 MHz and 8440 MHz. Moreover, $\mu_{r}$ was around 8439.01 MHz and the $\sigma_{r}$ was around 0.72 MHz. In Figure 5c, the blue curve represents the probability density of measured data and the red curve is the Gaussian curve with the same mean and variance as the experimental data. In order to further display the relationship between Figure 5b,c, the coordinates in the red box in Figure 5b indicate that it is in the corresponding region of half the Gaussian curve probability density. As such, the frequency mean value obtained by the Gaussian curve is displayed in Figure 5b with a red line. The data coordinates out of the red box, which we called “dirty data”, affected the mean value and increased the variance. We concluded that the phenomenon of frequency fluctuation often occurred in the OCMI system and affected the frequency mean, which lead to an inaccurate strain measurement.

**Figure 6:**
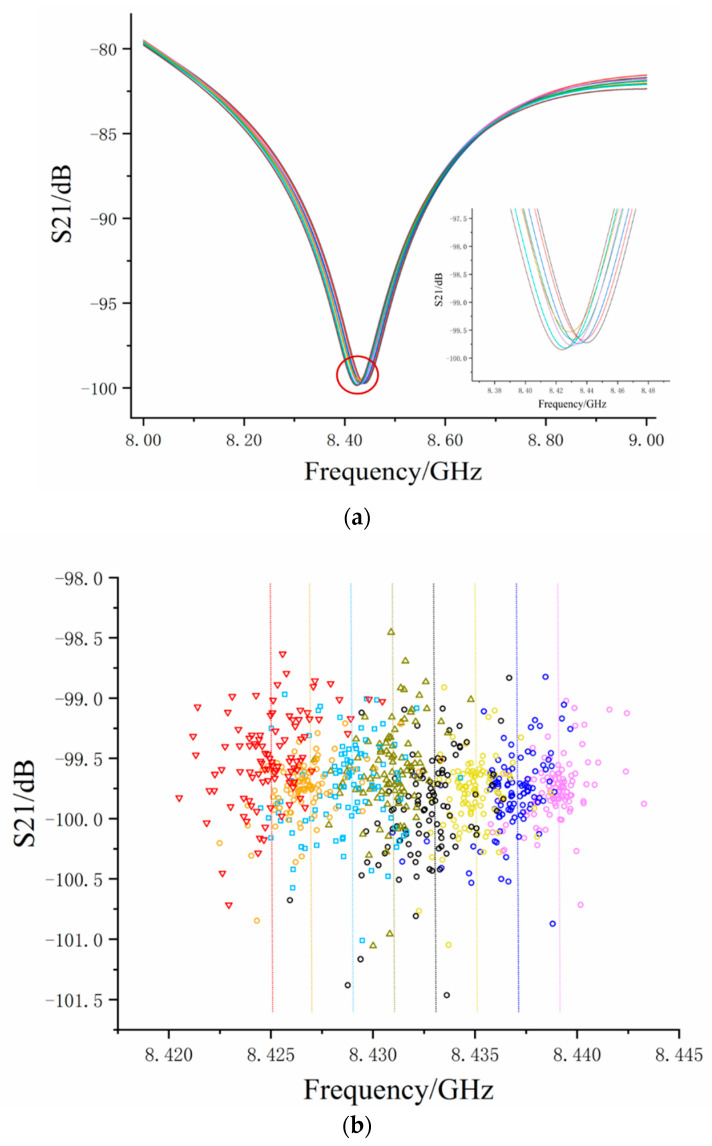
(**a**) The frequency shift of the microwave interference signal under strain changes. (**b**) Coordinates of S-parameter valley point of measured data.

After $\mu_{r}$ and $\sigma_{r}{}^{2}$ were obtained through $S_{TS}\left(x\right)$ in advance, we performed strain experiments on the strain test bench, where axial strains of 240 με per step were applied to the sensor by using a screw micrometer to pull the fiber. We stretched seven times and recorded 100 sets of data each time, so there were 800 sets of measured data $S_{WN}(x)$. As the strain increased, the OPD in the sensor increased and the interference spectrum moved toward a low frequency. Figure 6a shows the interference fringes at different strains during increasing steps. Through zoom in the valley of the S-parameter, we can clearly see the frequency shift. The measured data shown in Figure 6a was randomly selected from each experimental group to show the trend of interference fringes with a strain change. The fringe moved towards low frequency when the strain increased, indicating that the sensor’s OPD also increased.

Figure 6b shows the valley coordinates of the S-parameter interference fringes for all measured data. In order to distinguish them easily, the coordinates under different strains are represented by different colors and properties. Theoretical frequency was calculated by Equation (3) under each strain and is shown in Figure 6b with dotted lines of corresponding colors. It can be seen from Figure 6b that the valley coordinates of the S-parameter’s distribution were not uniform and the frequency drifted under the same strain because of electrical delay noise W and phase noise φ. A large number of dirty data were far away from the dotted line and some were even in the other strain state. For example, when the applied strain was 720 με, the measured data were black (Figure 6b). It can be seen that some valley coordinates were in the area of applied strain, 960 με, and some were in the area of applied strain 480 με. These dirty data greatly affect the mean value when the measured data involved in the estimation was insufficient and different levels of ‘dirty data’ led to large fluctuations in the estimated results the estimation results.

**Figure 7:**
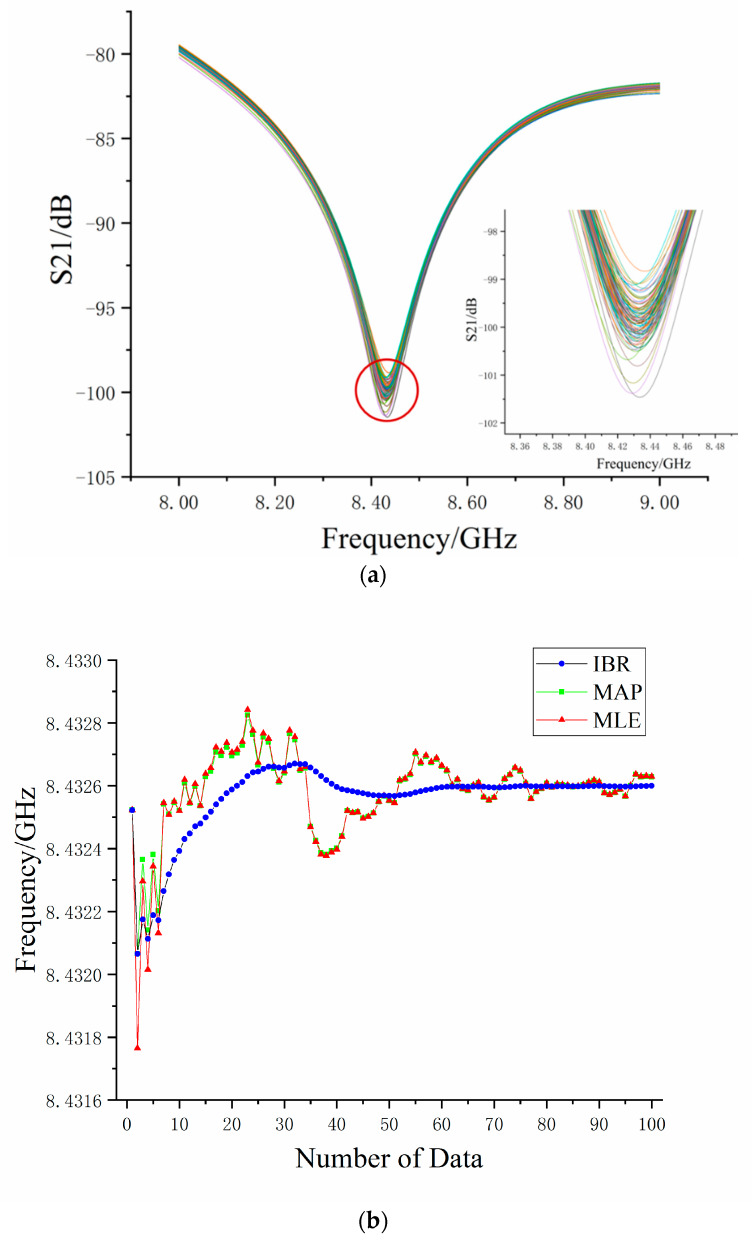
(**a**) 100 measured data of interference fringes for the S-parameter at 720 με strain. (**b**) Estimated frequency at the valley of measured data.

In order to explain the IBR algorithm for strain sensing in the OCMI, we used the groups of measured data with the largest frequency fluctuation to illustrate the process. Similar to the simulation, the MLE algorithm was used for comparison. We added the MAP algorithm for comparison to make the advantage of the IBR algorithm more evident. Figure 7a shows 100 measured data of interference fringes for the S-parameter with an applied strain at 720 με, whose S-parameter interference has a frequency fluctuation. The theoretical frequency was 8432.60 MHz, according to Equation (3), but the frequency shift to 8425.91 MHz was its worst. The estimated parameter $\mu_{n}$ and $\sigma_{n}$ of measured training sample $S_{TA}(x)$ were 8425.52 MHz and 1.28 MHz, respectively. We obtained a high accuracy and reliability result by iteratively rebalancing the weight between the estimated parameter and measured data.

Figure 7b shows the curve of the estimated value of the S-parameter valley point’s frequency via the IBR, MAP, and MLE algorithm with a number of measured data. It can be found from Figure 7b that the estimation results of three algorithms began at 8425.52 MHz and dramatically decreased when the measured data volume increased to three sets due to dirty data. The estimated results of both algorithms fluctuated up and down with the increase of measured data volume, finally stabilizing around 8432.60 MHz. The final estimated result was used as a baseline to analyze algorithms. Because the mean value was affected by dirty data, the result curve estimated via the MLE algorithm fluctuated greatly. The maximum frequency deviation was 0.8 MHz, which corresponded to 96 με, according to Equation (3). The MAP algorithm’s estimation results were consistent with the MLE’s results, except that the fluctuation was smaller than the MLE algorithm before 10 sets of data. However, for the IBR algorithm, the maximum frequency deviation was 0.4 MHz (48 με) and the curve of the estimated IBR results became smooth when the number of measured data increased to 10 sets. It did not fluctuate due to the one dirty data, as it did in the MLE.

Using the IBR algorithm, we can get a more accurate estimation at the initial stage (around 20 sets of measured data). Since the evolution curve of the estimated IBR results began to get gentle at around 20 sets of measured data, we reasonably regarded the estimated results by 20 groups of measured data as a stable value. The optimal results of the IBR algorithm for the measured data with applied strain at 720 με showed that the IBR optimized the mean effect of the dirty data and made the frequency estimation of the S-parameter closer to the true value. Compared to MLE and MAP, IBR used only a small amount of data to achieve the estimation result, reaching a state of relative stability and high reliability. In the process of iteration, the IBR algorithm rebalanced the weight of the prior information and data samples, making the prior information closer to the true value. At the same time, it made its weight larger and the estimation result more stable. Therefore, we estimated the results of 20 groups via the IBR algorithm to replace the estimated results of the MLE algorithm.

**Figure 8:**
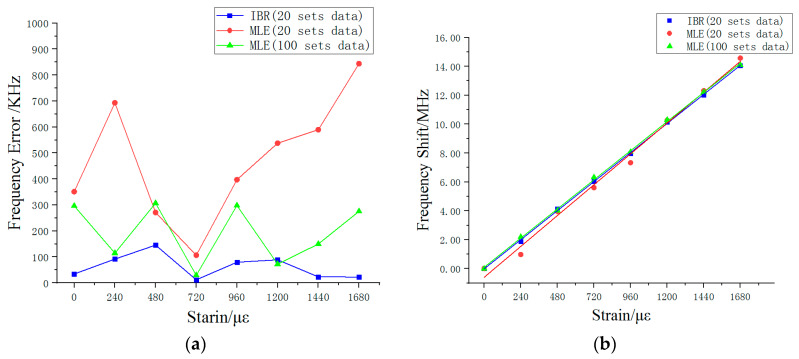
(**a**) The error rate of estimated results in each strain. (**b**) The estimated results and linear fitting by different sets of data.

In order to show the advantages of the algorithm, we took 20 groups of measured data under each strain and processed them with the IBR and MLE, respectively. We also took the estimated results of the MLE algorithm with 100 measured data under each strain as a comparison to IBR with 20 measured data. Figure 8a shows the frequency error between the theoretical frequency and estimated results for each strain. It can be seen that the MLE’s frequency error in 20 sets of data fluctuate largely. The maximum frequency error was 844 KHz and the average error rate was 25%. As the amount of data increased to 100 groups, its frequency error was optimized (around 200 KHz), with an average error rate of 9%. However, the IBR’s estimated results showed small frequency error (around 100 KHz) and the average error rate was only 3%. Figure 8b shows the relationship between strain and frequency shift obtained using the OCMI system over a large strain range and linear fitting. In the presence of large amounts of data (i.e., 100 sets of data for each strain), the final estimated value obtained using the MLE algorithm met the linear relationship in the graph. The frequency change of 1 MHz corresponded to 119 με, which was in line with the theoretical value 120 με calculated by Equation (3). The linear fitting degree of the estimated value processed using the MLE algorithm was 0.999. The frequency shift threshold for each strain change was 1.8–2.2 MHz (216–264 με). In the case of only 20 data sets, the estimated results of the MLE were unsatisfactory. The relationship between frequency and strain is shown in the Figure 8b as 1 MHz changed in corresponded to 115 με. Although its linear fitting degree was 0.991, the frequency shift changed each time, with a frequency shift threshold between 0.9–3.0 MHz (102–360 με). However, contrary to the MLE, the IBR algorithm performed well with 20 measured data sets. Figure 8b shows that the 1 MHz frequency change corresponds to the strain 119 με. The linear fitting degree of the IBR algorithm was 0.999, which was the same as the MLE in the presence of 100 sets of measured data. Moreover, the estimated results for each applied strain change tended to be more stable than found by the MLE, with a frequency shift threshold of 1.8–2.3 MHz (216–276 με).

On the basis of these results, we can conclude that the estimated result of the IBR is very reliable in a small amount of data (i.e., 20 data sets), which can be seen as the estimated result of MLE in a large amount of data (i.e., 100 data sets). The results demonstrate that the IBR algorithm obtains accurate experimental results through a small amount of data in the OCMI system. We believe that this method can be applied to many different environments besides the OCMI system, where the results measured will be affected by noise and its accuracy can improve through multiple measurements such as the distributed Raman fiber temperature measurement system and optical frequency domain reflectometer. Both systems need to get a set of accurate data through a large number of repeated experiments. The method can also be used for data processing, such as the geological data collected using the optical fiber sensor, which estimates and classifies the IBR algorithm to predict the seismic wave.

## 5. Conclusions

In this paper, we present a novel IBR algorithm based on the Bayesian theory for accurate results using only a few times measurements in the presence of noise, which reweighs the measured data and the estimated prior parameters in an iterative way to improve the S-parameter’s estimated valley point frequency. We verified the algorithm via simulation and compared it with the MLE in strain sensing experiments. The IBR algorithm only used 20 measured data to achieve the estimated results of MLE with 100 measured data. Using the same amount of measured data, the maximum strain deviation is reduced from 138 με to 36 με, and the average error rate decreases from 25% to 3%. The linear fitting degree of the estimated results of IBR reached 0.999. Unlike the MLE, which only arrived at an accurate estimated value after receiving more data, the IBR can obtain accurate and stable results. Because of the IBR method’s inherent complexity, it may be slower than MLE in calculation speed. Yet considering the OCMI system interference fringe frequency fluctuation, the algorithm can estimate the value of frequency under the influence of system noise. It is very flexible and adapts to different models of application in the OCMI system. Moreover, it is useful for the system with frequency fluctuation problem. A primary advantage of the IBR algorithm is the feasibility of stable estimated results without eliminating the possibility of sudden and large changes in value. Therefore, researchers can obtain accurate sensing data to measure strain changes by measuring only a few times, which greatly reduces the workload.

## References

[1] Capmany J., Novak D. (2007). Microwave photonics combines two worlds. Nat. Photonics.

[2] Jie H., Lan X., Wang H., Yuan L., Xiao H. Optical carrier based microwave interferometers for sensing application. Proceedings of the SPIE 9098.

[3] Wang Y., Wang M., Xia W., Ni X. (2016). High-resolution fiber Bragg grating based transverse load sensor using microwave photonics filtering technique. Opt. Express.

[4] Xiao L., Sun W., Veeraraghavan M., Hu W. (2017). Slotted store-and-forward optical circuit-switched networks: A performance study. IEEE/OSA J. Opt. Commun. Netw..

[5] Zhang J., Dong M., Wang D., Ye J., Jin Y. (2013). Construction of quasi-cyclic low-density parity-check codes for simplifying shuffle networks in layered decoder. Chin. Commun..

[6] Huang J., Lan X., Song Y., Li Y., Hua L., Xiao H. (2015). Microwave Interrogated Sapphire Fiber Michelson Interferometer for High Temperature Sensing. IEEE Photonics Technol. Lett..

[7] Camparo J.C., Coffer J.G. (1999). Conversion of laser phase noise to amplitude noise in a resonant atomic vapor: The role of laser linewidth. Phys. Rev. A.

[8] Woodward S.L., Stayt J.W., Romero D.M., Freund J.M., Przybylek G.J. (1998). A study of optical beat interference between Fabry-Perot lasers. IEEE Photonics Technol. Lett..

[9] Wang A., Gollapudi S., Murphy K.A., May R.G., Claus R.O. (1992). Sapphire-fiber-based intrinsic Fabry—Perot interferometer. Opt. Lett..

[10] Rüdiger P. (2009). Fiber Amplifiers—Part 2.

[11] Patel K., Negi P.S., Kothari P.C. (2009). Complex S-parameter measurement and its uncertainty evaluation on a vector network analyzer. Measurement.

[12] Moose P.H. (1994). A technique for orthogonal frequency division multiplexing frequency offset correction. IEEE Trans. Commun..

[13] Lin D.D., Pacheco R.A., Lim T.J., Hatzinakos D. (2006). Joint estimation of channel response, frequency offset, and phase noise in OFDM. IEEE Trans. Signal Proc..

[14] Zou Q., Tanghat A., Kim K.Y., Sayed A.H. OFDM channel estimation in the presence of frequency offset, IQ imbalance and phase noise. Proceedings of the IEEE International Conference on Acoustics, Speech & Signal Processing.

[15] Balogun M.B., Oyerinde O.O., Takawira F. Performance of ML-based carrier frequency offset estimation in CO-OFDM systems. Proceedings of the 2017 IEEE Africon.

[16] Balogun M.B., Oyerinde O.O., Takawira F. (2019). Simplified ML-based Carrier Frequency Offset and Phase noise estimation for CO-OFDM systems. SAIEE Afr. Res. J..

[17] Halimi A., Buller G.S., McLaughlin S., Honeine P. (2016). Bayesian filtering of smooth signals: Application to altimetry. arXiv.

[18] Sanger T.D. (2006). Bayesian Filtering of Myoelectric Signals. J. Neurophysiol..

[19] Xiang Y., Akcakaya M., Sen S., Erdogmus D., Nehorai A. (2019). Target tracking via recursive Bayesian state estimation in cognitive radar networks. Signal Proc..

[20] D’Agostini G. (2003). Bayesian inference in processing experimental data: Principles and basic applications. Rep. Prog. Phys..

[21] Box George E.P., Tiao George C. (1973). Bayesian Inference in Statistical Analysis, Addison-Wesley Series in Behavioral Science: Quantitative Methods.

[22] Karlsson R., Gustafsson F. (2005). Recursive Bayesian estimation: Bearings-only applications. IEE Proc. Radar Sonar Navig..

